# Training academic staff for effective feedback in workplace-based assessment: a study in Bhutan

**DOI:** 10.1186/s12909-025-07314-4

**Published:** 2025-05-22

**Authors:** Sontosh Mukhia, Karma Tenzin

**Affiliations:** 1 Department of ENT surgery, Jigme Dorji Wangchuck National Referral Hospital, Thimphu, Bhutan; 2 School of Medicine and Public Health, University of Newcastle, Newcastle, NSW Australia

**Keywords:** Faculty development training, Feedback, Perception, Workplace-based assessment

## Abstract

**Introduction:**

The feedback plays a critical role in competency-based education in both undergraduate and Postgraduate medical education. The study explores the impact of a faculty development program on feedback practices of residents and faculty of ENT and ER medicine at Gyalpo University of Medical Sciences of Bhutan (KGUMSB).

**Methods:**

This mixed method study was conducted in two departments with 14 faculty members participating in the study. The questionnaire was used to obtain the perception of feedback before and after a Faculty Development Training (FDT) on good feedback practices. Student “t” test was used to compare the feedback perception at day 0 and 6 months and the responses were qualitatively analyzed using thematic analysis.

**Results:**

*(a) Quantitative*: The confidence of faculty to provide feedback improved significantly after FDT as compared to before FDT and it persisted in the same for 6 months (*p*-value 0.041 and *p*-value 0.027 respectively). The overall perception of feedback as a tool significantly changed positively after FDT and at 6 months (*p*-value *p*-value = 0.000). *(b) Qualitative*: Two thematic areas of process and teaching-learning were analyzed. Faculty showed improved and more focused feedback after training, but signs of decline by 6 months highlighted the need for refresher training. Feedback initially improved for residents, as it became more constructive and useful, though by 6 months, it showed potential for further refinement and consistency.

**55 -Conclusions:**

The findings from this study are suggestive that feedback may have excellent potential as a tool for enhanced student learning in WPBA encounters.

**Supplementary Information:**

The online version contains supplementary material available at 10.1186/s12909-025-07314-4.

## Introduction

Faculty of Postgraduate Medicine, Khesar Gyalpo University of Medical Sciences of Bhutan established in 2014, introduced workplace-based assessment tools namely Direct Observations of Procedural Skills (DoPS), Mini-Clinical Evaluation Exercise (Mini CEX), Case-based discussion (CbD) and multi-soure feedback in postgraduate training in 2018 [1, 2]. Detailed and timely feedback on performance is central to competency-based and outcome-based medical education [3]. The residents are mandated to complete at least three WPBA in each of their rotations to enhance their learning.

However, feedback effectiveness is lowered if perceived as a threat to self-esteem if focus is on a person rather than a person’s professional behavior. Such feedback makes teaching-learning ineffective [4, 5]. It is of paramount importance to have sufficient feedback giving and receiving skills to enhance learning [6, 7]. Therefore, faculty development training to provide effective feedback is critical in improving the educational process [8].

Feedback is a supportive conversation that is focused mainly on the trainee’s progress by focusing on developing competencies, enhancing self-efficacy, challenging them to set objectives for improvement and facilitating their development of strategies [5, 9–11]. It serves as an informal assessment tool that is provided immediately following a direct observation to achieve their best potential [12]. Effective feedback, therefore, should be routine, timely, non-threatening, specific, encourage self-assessment, working on required learning and setting performance goals during next encounter [11, 13].

There was a perceived awareness that the feedback literacy was low among the faculty members at KGUMSB. This was mainly since a new faculty is recruited based on certificate secured from an online course with no face-to-face practical session. So, it was felt that faculty development training on effective feedback practices was much needed.

This study aims to explore how training influences faculty confidence in providing effective feedback, its impact on teaching-learning, and residents’ perceptions.

## Methods

### Study design, site and period

The study was a mixed-method study. The Research Ethics Board of Health provided ethical approval for the study (Ref. No. IRB/Approval/PN21-025/2021/519, dated: 30/11/2021), Khesar Gyalpo University of Medical Sciences of Bhutan. The study was conducted at the ENT and Emergency Medicine department at Jigme Dorji Wangchuk National Referral Hospital in Thimphu from March 2022 to March 2023. Written informed consent was obtained from all participants of the study.

### Study population and sample size

All the faculty members and existing postgraduate residents were included in the study. A total of seven faculty (5 from the Department of Otolaryngology-Head and Neck Surgery-ENT and 2 from the Department of Emergency Medicine-ER) and seven residents (2 from ENT and 5 from ER) were enrolled for the study after getting informed of written consent.

### Questionnaire

Two separate questionnaires were developed for faculty and residents. The resident questionnaire included 10 questions on their experience with feedback encounter and similarly, there were 11 questions in the faculty questionnaire for capturing their experience with feedback session.

The questionnaire was validated by 3 local medical education experts for content, language and relevancy of the questions and for the time required to fill in the questionnaire. It was then piloted tested among the faculty members and residents from the departments who were not involved in the study. Minor language refinement was done following the feedback on the questionnaire.

### Description of intervention

#### Step 1. sensitization

A half-day faculty development workshop was organized for all 14 participants where the basic concepts, confirming and disconfirming, rules of giving and receiving feedback were introduced. The faculty members were trained on good feedback practices through lectures, videos and roles plays. In addition, they were also provided with practice to provide and receive feedback with just-in-time inputs from the facilitators. The reason for including only two departments was based on two aspects, firstly there was a feasibility issue of bringing all facility from various departments under one roof at the same time as there are shift systems and not everyone is at the station at the same time. The second was that in most of the Departments there are only single or at the most 2 residents with almost equal number of faculty members. This explains why only 2 departments were attempted for this study, along with small sample size.

#### Step two

A pre-validated questionnaire was utilized to assess feedback practices after WPBA encounters. It included six closed-ended questions on clarity, usefulness, behavior change, competency reinforcement, motivation, and satisfaction. Faculty also answered an open-ended question on confidence in providing feedback. Responses were collected using a 0–10-point Likert scale, with 10 indicating the highest agreement. Both faculty and residents reflected on the feedback encounters.

#### Step three

Following the evaluation of existing practice. A one-day training on good feedback practices was conducted for the seven faculty members at Khesar Gyalpo University of Medical Sciences by competent trainers (FAIMER graduates). The faculty members were trained on good feedback practices through lectures, videos and role plays.

#### Step four:data collection process

**Fig. 1:**
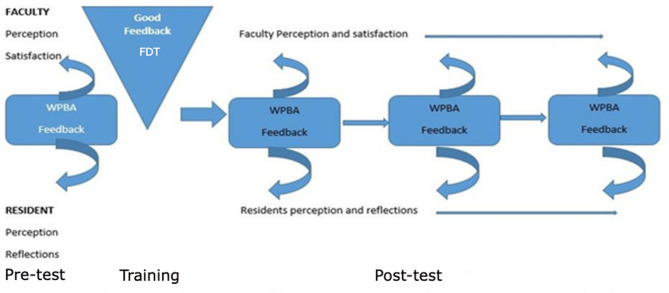
The data collection process

Data was collected using a self-administered questionnaire in paper or Google Forms immediately after a WPBA feedback encounter, which is one to one setting between a single faculty and single resident. Participants reflected on the usefulness, clarity, expected behavioral change, competency reinforcement, motivation, and satisfaction. Faculty also rated their confidence in providing feedback. This data was gathered four times using the same questionnaire; before the faculty development training, within 2 months of FDT, 2–4 months, and 4–6 months afterward as shown in Fig. 1.

#### Step five

For everyone encounter, a resident received feedback from a single faculty (assessor). This feedback was conducted in a conducive and friendly environment in a separate room away from patients and other health professionals. In the end, each resident was given time to reflect on the WPBA encounter and make a note.

### Data analysis

#### Quantitative component

The data were organized into responses collected day zero, within 2 months, 2–4 months, and 4–6 months after the intervention, then analyzed using SPSS v26.

A Student t-test compared feedback on faculty confidence, usefulness, clarity, expected behavior change, competency reinforcement, motivation, and satisfaction across these periods.

#### Qualitative component

The reflective responses after each feedback were divided into each period and a thematic analysis was done. Codes and themes were inductively derived using free qualitative data analysis software- QDA lite miner version 6.

## Results

The response rate was 100% with all enrolled participants completed the data collection process by filling up the questionnaire at 0, 2 months, 4 months and 6 months.

### Quantitative results

The faculty members reported that by the end of 6 months they became more confident, and better understood the usefulness of the feedback, it was perceived that they were able to motivate the residents better and then finally their satisfaction level also increased compared to the pre-intervention period as shown in Table 1.

**Table 1:** Mean score of perceptions of faculty and residents over different months

Sl. No	Questions	Mean score before FDT *n* = 7	Mean score after FDT *n* = 7		
A	**Feedback questions to the faculty**	At 0 month	0–2 months	2–4 months	4–6 months
1.	C**onfident** to give feedback	5.57 ± 1.27	8.25 ± 0.5	7.50 ± 1.18	7.33 ± 1.22
2.	How **useful** do you think the resident found your feedback?	5.86 ± 0.69	7.75 ± 0.05	7.30 ± 1.25	7.33 ± 0.86
3.	C**learly** of your feedback.	6.43 ± 0.98	8.25 ± 0.50	7.30 ± 1.06	7.44 ± 1.14
4.	Chance of residents **changing** his/her behavior after your feedback.	6.43 ± 0.97	8.25 ± 0.96	7.50 ± 1.08	7.78 ± 0.83
5.	Your level of **reinforcement on** his/ her competency on the current performance	6.57 ± 1.39	8.50 ± 0.57	7.60 ± 1.07	7.56 ± 0.72
6.	Your feedback **motivated** the residents to perform better in future?	7.14 ± 0.90	8.25 ± 0.50	7.40 ± 1.07	7.78 ± 1.20
7.	your **satisfaction** after the feedback session	6.14 ± 1.34	8.75 ± 0.50	7.50 ± 1.17	7.67 ± 1.11
B	**Feedback questions to the resident**	At 0 month	0–2 months	2–4 months	4–6 months
1.	The feedback Was useful	7.14 ± 1.07	7.70 ± 1.57	7.0 ± 2.00	7.88 ± 1.25
2.	The feedback clearly presented and structured	6.43 ± 1.27	7.10 ± 2.13	6.20 ± 1.30	7.38 ± 1.50
3.	The feedback you have received will **change** the way you approach future encounter.	7.14 ± 0.90	8.10 ± 1.96	6.80 ± 1.92	8.00 ± 1.19
4.	The feedback **reinforced my** competency regarding the current task.	7.00 ± 1.00	8.20 ± 1.93	7.00 ± 2.12	7.88 ± 1.24
5.	How **satisfied** are you with this encounter?	7.00 ± 1.15	8.00 ± 2.21	7.20 ± 1.78	7.88 ± 1.55

Similarly, residents reported that by the end of 6 months, they saw feedback as a useful tool for enhancing learning through clarity, reinforcement, motivation and satisfaction as their perception about feedback increased compared to the pre-intervention period as shown in Table 1.

**Fig. 2:**
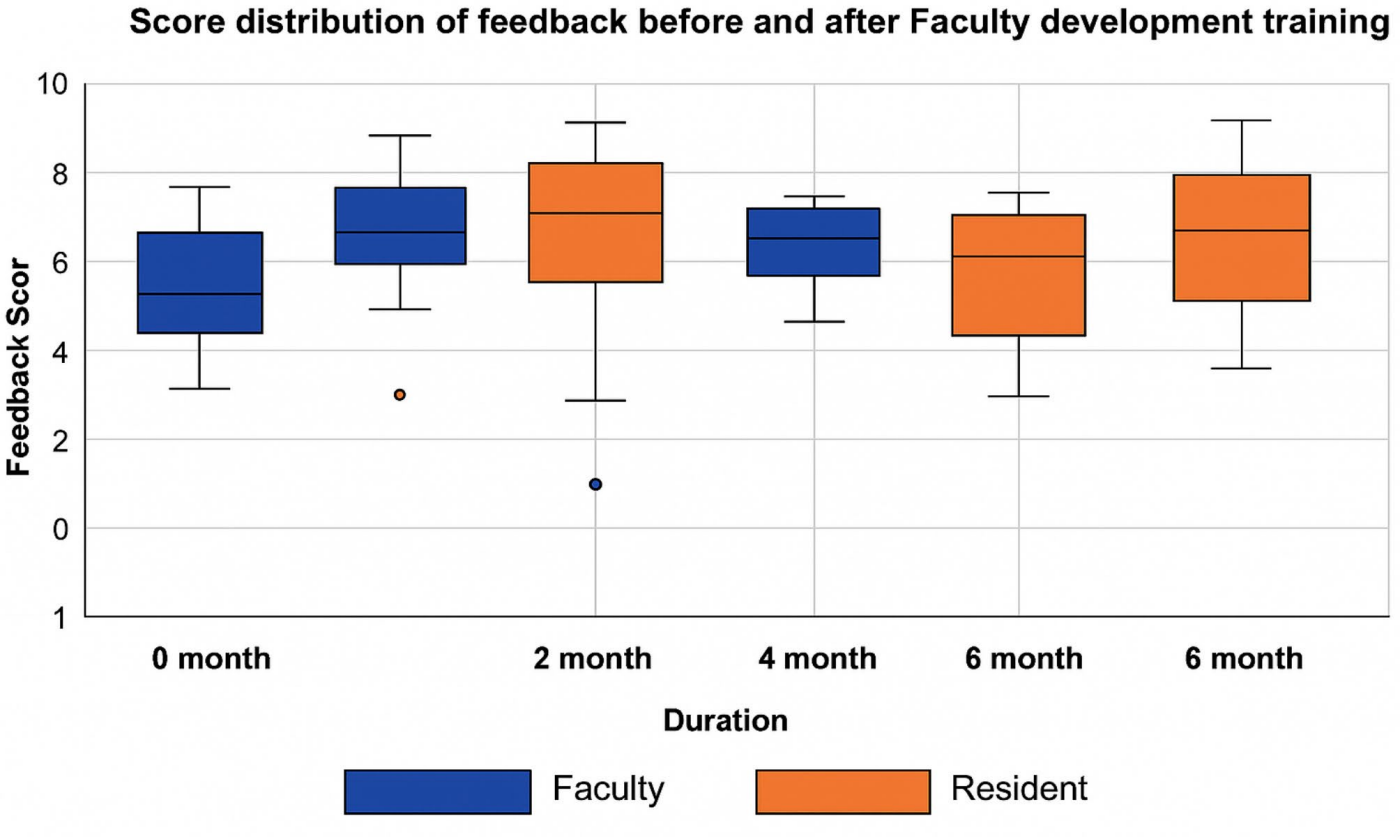
Mean score distribution of overall feedback perception before and after FDT

**Table 2:** Faculty’s perception on feedback over different periods

Faculty’s perception	Before FDT and 2 months *n* = 7		2 months and 6 months *n* = 7		Before FDT and 6 months *n* = 7	
	*p* value	95% CI	*p* value	95% CI	*p* value	95% CI
*Confidence*	**0.007**	3.25	0.23	1.25	**0.032**	1.85
		(1.72–4.77)		(1.46–3.96)		(0.21–3.43)
*Usefulness*	**0.018**	2.25	0.182	0.50	0.134	1
		(0.72–3.70)		(0.41–1.41)		(0.41–2.41)
*Clarity*	**0.003**	2.25	0.319	0.75	0.078	0.857
		(1.45–3.04)		(1.25–2.75)		(0.132–1.84)
*Expected*	0.058	2.25	0.638	0.25	**0.008**	1.42
*Behavioral*		(0.13–4.63)		(1.27–1.77)		(0.52–2.330
*change*						
*Competency*	0.063	2.5	0.391	0.5	0.103	1.14
*reinforcement*		(0.25–5.2)		(1.09–2.09)		(0.31–2.59)
*Motivation*	**0.014**	1.5	0.761	0.25	0.134	1
		(0.58–2.41)		(2.13–2.63)		(0.413–2.41)
*Satisfaction*	**0.001**	3.25	0.495	0.50	**0.045**	1.71
		(2.45–4.04)		(1.55–2.55)		(0.05–3.37)

* Student t-test was performed

Following the Faculty Development Training (FDT) on effective feedback, faculty confidence in giving feedback significantly improved (*p* = 0.007) and remained stable at 2, 4, and 6 months.

Perceptions of feedback usefulness, clarity, motivation of residents, and overall satisfaction also improved significantly after the FDT. These remained consistent over time, except for satisfaction and expectations of resident behavioural change, which showed further significant improvement at 6 months (*p* = 0.045 and 0.008, respectively). (See Table 2.)

**Table 3:** Residents’ perception on feedback over different periods

Resident’s Perception	Before FDT and 2 months *n* = 7		2 months and 6 months *n* = 7		Before FDT and 6 months *n* = 7	
	*P* value	95% CI	*P* value	95% CI	*P* value	95% CI
*Usefulness*	0.887	0.143	0.634	0.375	0.341	0.857
		(2.29–2.49)		(1.40–2.59)		(1.17–2.88)
*Clarity*	0.582	0.714	0.895	0.125	0.139	1.14
		(2.29–3.72)		(2.03–2.28)		(0.49–2.78)
*Expected*	0.611	0.57	1	0.00	0.216	1
*Behavioral*		(2.03–3.17)		(2.04–2.04)		(0.77–2.77)
*change*						
*Competency*	0.550	0.71	0.89	0.125	0.216	1
*reinforcement*		(2.01–3.47)		(1.94–2.19)		(0.77–2.77)
*Motivation*	0.587	0.57	0.89	0.125	0.27	0.85
		(1.86–3.01)		(2.08–2.33)		(0.67–2.58)
*Satisfaction*	0.75	0.42	0.90	0.125	0.30	1 (1.2–3.2)
		(2.72–3.58)		(2.38–2.63)		

* Student t-test was performed

Residents on the other hand had a higher mean score compared to the faculty’s score at 0 months as shown in Fig. 2. Although at 2 months after FDT the mean score improved, there was no significant change in residents’ perception of feedback being useful, clarity of feedback, expected behavioral change, competency reinforcement, motivation, and satisfaction after the WPBA encounters as shown in Table 3.

**Table 4:** The overall perception of feedback of faculty and residents over different periods

Overall Perception	Before FDT and 2 months		2 months and 6 months		Before FDT and 6 months	
	*p* value	95% Confidence interval	*p* value	95% Confidence interval	*p* value	95% Confidence interval
***Faculty’s*** ***perception***	**0.00**	2.12 (1.67–2.57)	**0.004**	0.75 (0.261.23)	**0.00**	1.19 (0.75–1.62)
***Resident’s***	**0.025**	0.78	1	0	**0.01**	0.90
***perception***		(O.10-1.48)		(0.678-0.678)		(0.401.41)

* Student t-test was performed

On the overall perceptions of faculty and residents, both the groups showed effective feedback and had a significant impact on teaching-learning after the FDT (*p*-value < 0.05). The faculty over the period of 6 months had scored overall feedback perceptions as significant. Whereas residents for the same period did not score overall perception of feedback as significant. However, as compared to before the FDI and at 6 months, the overall impact on teaching and learning remained significant. Table 4.

### Qualitative results

**Fig. 3:**
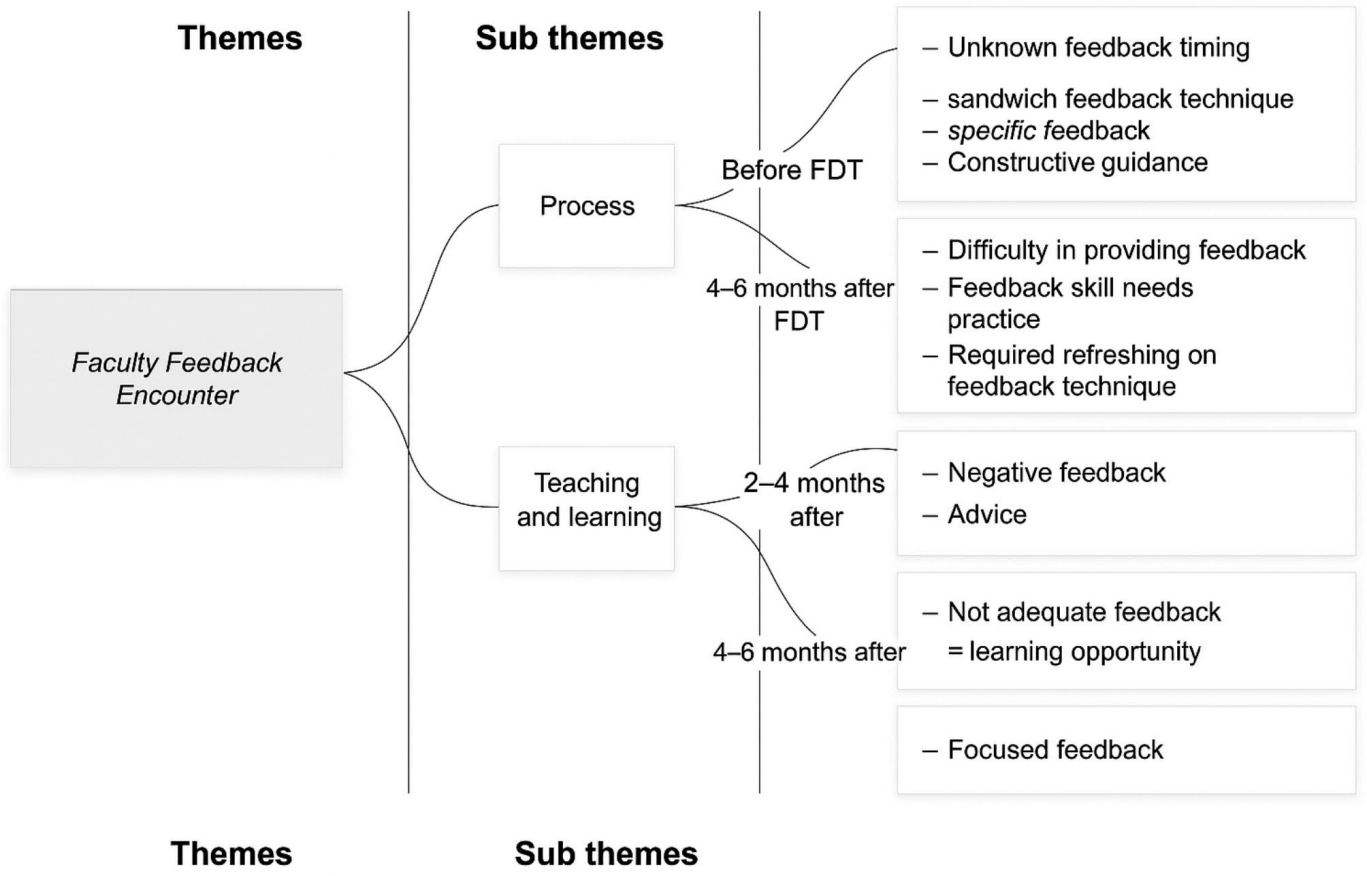
Thematic representation of Faculty on Feedback provided to the residents

**Fig. 4:**
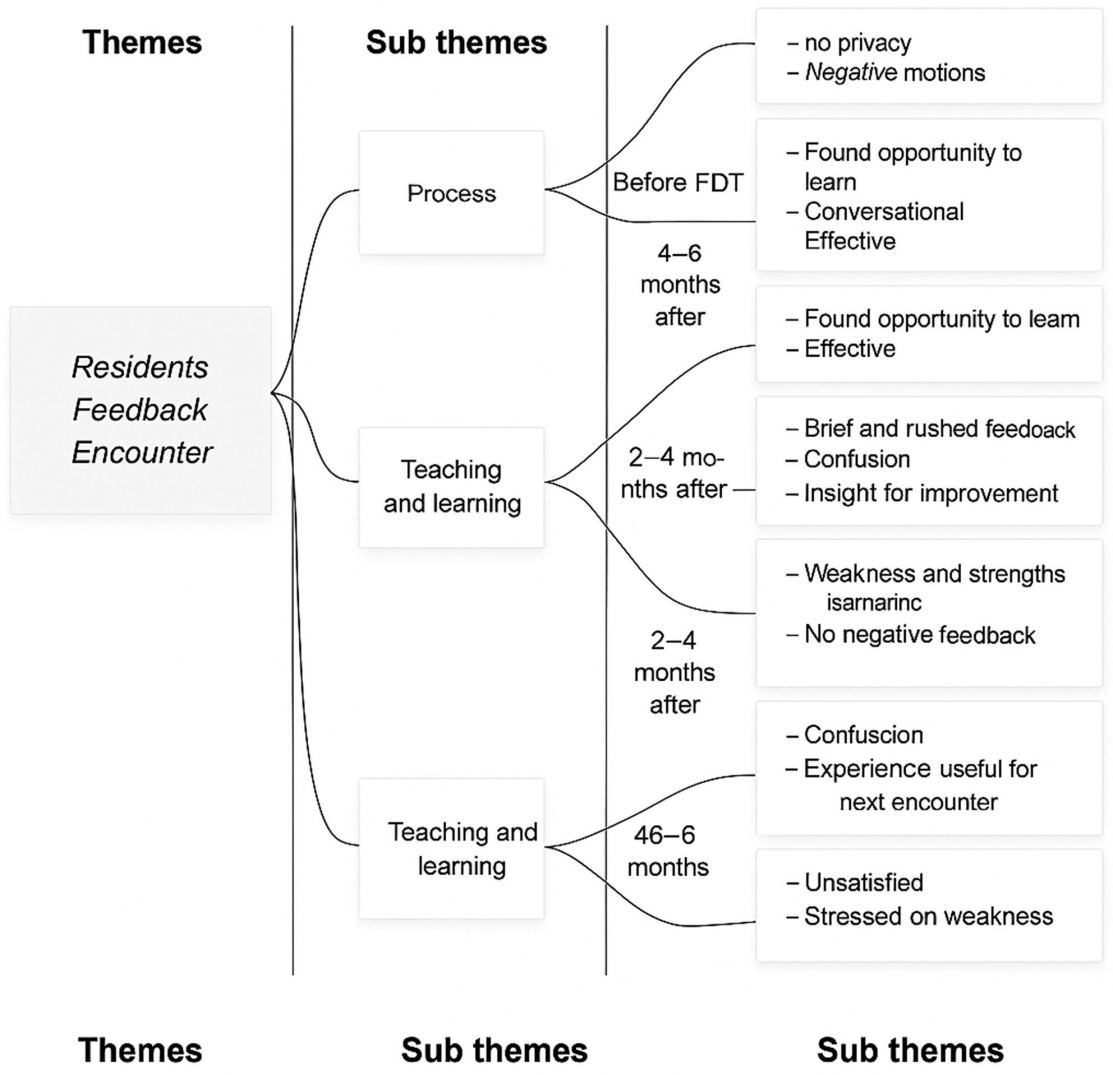
Thematic representation of Residents on Feedback received

Qualitative data were collected as a reflection of the feedback encounters. It was also collected before the faculty development training, within two months, within 2–4 months and within 4–6 months. QDA miner lite version 5 was used to analyze open-ended questions. Inductively derived codes were used to develop subthemes and themes as represented in Figs. 3 and 4.

### Comparison of qualitative and quantitative results

#### Before and within 2 months after FDT

There was a statistically positive change in confidence and perceptions of both faculty and resident after FDT. Before FDT, faculties were disrupting the process of WPBA and feedback was not specific. Residents on their part felt humiliated and shy but were determined to do better and improve next time.

R1: “I need to learn, practice and be competent.”

Residents preferred receiving meaningful feedback from encounters [14], consistently scoring higher without significant change over 2, 4, and 6 months. Following FDT, faculty employed various feedback models with a greater focus on residents’ learning. Appropriately planned, descriptive feedback proved effective, accurate, and educational.

(R2) One resident remarked, “*The feedback was effective and accurate*,* focusing on strengths and weaknesses*.”

#### 2 Months to 6 Months after FDT

There was significant overall improvement in perception of feedback in both faculty and resident. However, only faculty at 6 months had significant positive change in feedback being usefulness, instilled an expected behavioral change and satisfaction. This was because a faculty realized shortcomings in his feedback process and reflected on the FDT.

*F3: “I had revised the good feedback practice literature and videos. It helped me this time to provide the resident with focused feedback and it enriched this teaching learning encounter.”*

Residents at 6 months had mixed opinions on their perception of feedback mainly because one of the faculty revised the good feedback practices. FDT longitudinally improves confidence and effectiveness of feedback.

*R5: “I was told that I would kill the patient in that case. I was not given the opportunity to clarify. The encounter was brief*,* and I am not satisfied.”*

## Discussion

This findings from this study suggest that both faculty and residents significantly changed their perception of feedback as a learning tool following faculty development training (FDT). Six months post-FDT, faculty confidence in providing feedback improved. For residents, perceptions of feedback clarity, competency reinforcement, motivation to learn, and satisfaction with feedback also saw improvements [15].

Studies, including one from Saudi Arabia reported up to 50% require faculty development training (FDT) to develop feedback competence. At KGUMSB, FDT for new teachers was established since 2017, which has improved their self-efficacy and teaching competency [16, 17]. The introduction of WPBA in July 2018, further normalised the feedback culture, with both faculty and residents viewing this as positive development. A similar improvements has been noted after a feedback workshop, reinforcing the value of structured training [18, 19].

Feedback proficiency plateaued 2–6 months post-FDT, highlighting the need for continued practice, which corelates with need for long-term faculty development supports sustained improvement [20, 21]. Residents, who view feedback as essential and are required to engage through WPBA, showed higher perception scores even before FDT as reported in other studies [1, 12, 18]. Numerous studies also emphasized the need to reinforce feedback skills, highlighting the importance of utilizing the best practices, reflecting on previous encounters, and always modifying feedback approaches to develop effectiveness [5, 22, 23].

The Qualitative analysis focused on two key thematic areas as impact of feedback on the process and teaching-learning. As reported by the many other studies [3, 8, 15], both process and the Teaching-learning improved initially which were observed in resident saying;

(R2) One resident remarked, “*The feedback was effective and accurate*,* focusing on strengths and weaknesses*.”

This matches with the quantitative data captured in Table 2.

The a novice faculty seemed unfocused and did not see any privacy issues while providing feedback which the students thought otherwise. After 6 months of consistent utilization of feedback session, the faculty were more focused however, student were unsatisfied. This is not uncommon perception as the same as has been reported by other researchers too (16, 19, 21 & 22).

*R5: “I was told that I would kill the patient in that case. I was not given the opportunity to clarify. The encounter was brief*,* and I am not satisfied”.*

The other plausible explanation for such remark by faculty is the amnesia onset due to long training gap or need for lower dose with high frequency training on feedback techniques as suggested by studies (20, 23, 14 & 24).

### Strengths and limitations

The study explores feedback practices following faculty training on effective feedback methods, providing valuable insights. The study was conducted in small size sample and conducted over a short period of time, this limits the generalizability of the findings.

## Conclusions

Training in effective feedback is indicative of enhanced student learning and boosted faculty confidence in giving feedback. The overall experience of feedback by both faculty and residents enhanced over a period. However, a saturation point was observed, signaling the need for continuing and frequent refresher training.

## Electronic supplementary material

Below is the link to the electronic supplementary material.

Supplementary Material 1

## Data Availability

Data is provided within the manuscript.
