# Supplementary material for: Training academic staff for effective feedback in workplace-based assessment: a study in Bhutan
Source: BMC Med Educ. 2025 May 22;25:748. doi: 10.1186/s12909-025-07314-4 (PMC12096605; doi:10.1186/s12909-025-07314-4)
Supplement: Supplementary file 1 — Supplementary Material 1 [file 12909_2025_7314_MOESM1_ESM.docx]

**ANNEXURE II:** Study Tool

**Questionnaire for Faculty**

**“Introduction of Faculty Development Training on good feedback practices for Workplace based Assessments and its impact on faculties and residents working at the ENT and Emergency Medicine of Khesar Gyalpo University of Medical Sciences, Bhutan”**

(Please call Dr. Sontosh Mukhia at 17777606 for any queries while filling up this form)

**Date:**

**WPBA type: DoPS / mini-Cex / CbD**

**Setting: OPD / ward / Classroom / other**

1. How **confident** are you in giving the feedback?

**1 2 3 4 5 6 7 8 9 10 HIGH**

1. How **useful** do you think the resident found your feedback?

**1 2 3 4 5 6 7 8 9 10 HIGH**

1. How **clearly** did you provide your feedback?

**1 2 3 4 5 6 7 8 9 10 HIGH**

1. How likely do you feel that the resident will **change** his/her behavior after your feedback?

**1 2 3 4 5 6 7 8 9 10 HIGH**

1. To what extent did you **reinforce** his/ her competency on the current performance?

**1 2 3 4 5 6 7 8 9 10 HIGH**

1. To what extent the feedback given by you **motivated** the resident to perform better in future?

**1 2 3 4 5 6 7 8 9 10 HIGH**

1. How **satisfied** are you after this feedback session?

**1 2 3 4 5 6 7 8 9 10 HIGH**

1. What is your **reflection** from this WPBA feedback session? (Describe briefly in few sentences)

**Questionnaire for Resident**

**“Introduction of Faculty Development Training on good feedback practices for Workplace based Assessments and its impact on faculties and residents working at the ENT and Emergency Medicine of Khesar Gyalpo University of Medical Sciences, Bhutan”**

(Please call Dr. Sontosh Mukhia at 17777606 for any queries while filling up this form)

Date:

WPBA type: DoPS / mini-Cex / CbD

Setting: OPD / ward / Classroom / other

1. Overall, how **useful** was the feedback you have just received on your WPBA encounter?

**1 2 3 4 5 6 7 8 9 10 HIGH**

1. How **clearly** has the feedback been presented and structured?

**1 2 3 4 5 6 7 8 9 10 HIGH**

1. How likely do you feel that the feedback you have received will **change** the way you approach future encounter?

**1 2 3 4 5 6 7 8 9 10 HIGH**

1. To what extent does the structured feedback you have just received **reinforce** your competency?

**1 2 3 4 5 6 7 8 9 10 HIGH**

1. To what extent will this feedback **encourage** you to undertake to ‘complete’ this task in the future?

**1 2 3 4 5 6 7 8 9 10 HIGH**

1. How **satisfied** are you with this encounter?

**1 2 3 4 5 6 7 8 9 10 HIGH**

1. What is your **reflection** from this WPBA feedback session? (Describe briefly in few sentences)
